# Adapting T Cell Receptor Ligand Discrimination Capability *via* LAT

**DOI:** 10.3389/fimmu.2021.673196

**Published:** 2021-04-16

**Authors:** Wan-Lin Lo, Arthur Weiss

**Affiliations:** ^1^ Division of Rheumatology, Rosalind Russell and Ephraim P. Engleman Arthritis Research Center, Department of Medicine, University of California, San Francisco, San Francisco, CA, United States; ^2^ Howard Hughes Medical Institute, University of California, San Francisco, San Francisco, CA, United States

**Keywords:** LAT, coreceptor scanning, LCK, CD8, PLCγ1, T cell receptor ligand discrimination, kinetic proofreading model

## Abstract

Self- and non-self ligand discrimination is a core principle underlying T cell-mediated immunity. Mature αβ T cells can respond to a foreign peptide ligand presented by major histocompatibility complex molecules (pMHCs) on antigen presenting cells, on a background of continuously sensed self–pMHCs. How αβ T cells can properly balance high sensitivity and high specificity to foreign pMHCs, while surrounded by a sea of self-peptide ligands is not well understood. Such discrimination cannot be explained solely by the affinity parameters of T cell antigen receptor (TCR) and pMHC interaction. In this review, we will discuss how T cell ligand discrimination may be molecularly defined by events downstream of the TCR–pMHC interaction. We will discuss new evidence in support of the kinetic proofreading model of TCR ligand discrimination, and in particular how the kinetics of specific phosphorylation sites within the adaptor protein linker for activation of T cells (LAT) determine the outcome of TCR signaling. In addition, we will discuss emerging data regarding how some kinases, including ZAP-70 and LCK, may possess scaffolding functions to more efficiently direct their kinase activities.

## Introduction

The immune system maintains a homeostatic state within an organism while remaining poised to vigorously respond to life-threatening challenges. T cells, as a major component of the adaptive immune system, contribute to this difficult task by maintaining a level of tonic signaling which is required for their survival under homeostatic conditions. However, these same T cells are capable of rapidly responding to challenges by pathogens or cancerous cells to induce remarkably precise immune responses through the generation of signals that lead to clonal expansion and acquisition of effector functions only by appropriate antigen-specific T cells.

The T cell antigen receptor (TCR) is the major surface receptor used by every T cell to survey host cells expressing short self-peptides derived from host self-proteins bound to self-MHC molecules (self–pMHC). At the same time, peptides derived from pathogen proteins that are likewise bound to MHC molecules can also be recognized by the TCR and serve as agonists to initiate T cell responses in a highly sensitive and specific manner. One to ten foreign agonist–pMHCs are sufficient to activate an antigen-specific T cell (thus, high sensitivity); each TCR clonotype also reacts with one foreign agonist–pMHC (thus, high specificity) (1, 2). However, a significant portion of the naive T cell repertoire is also capable of responding to multiple foreign antigens (thus, cross-reactivity) or to allogeneic-MHC complexes (alloreactivity) (3, 4).

## T Cells Need to Properly Discriminate Among Foreign, Self-, and Absence of Peptide–MHC Stimulation

Circulating naive T cells continuously survey their local environments with their TCRs *via* interactions with self–pMHCs which generates survival signals, so called tonic signaling (5–10). Those naive T cells that fail to engage self–pMHCs rapidly die (11, 12). The levels of tonic signaling received by each individual T cell varies from clone to clone, and are correlated with each TCR’s reactivity towards particular self–pMHCs (13, 14). Importantly, the TCR’s reactivity toward self–pMHCs also influences the functional potential of T cells during anti-bacterial or anti-viral immune responses (13, 15–19), or dictates regulatory T cell suppressive function (20). These data suggest that tonic signaling plays an active role in modulating or adapting the ability of T cells to mediate effector functions. How T cells can achieve such precise immune regulation while avoiding autoimmune diseases remains unclear. More specifically, how do T cells properly discriminate a foreign antigen from the sea of self–pMHCs, and discriminate self–pMHC stimulation from the absence of pMHCs?

T cell ligand discrimination instructs fate decisions at multiple developmental, homeostatic and differentiation stages. The basis for the molecular mechanisms underlying T cell ligand discrimination is an important question, especially in the context of recent advances in T cell-based immune therapies. For example, chimeric antigen receptor (CAR)-induced signaling requires more ligand-binding events and is less sensitive than natural/conventional TCR signaling (21–23). Such therapy can also lead to off-target antigen recognition driven T cell expansion and inflammation in healthy tissues (24–26). A better fundamental understanding of how T cells discriminate self- from non-self-ligands undoubtedly will facilitate the improvement of current T cell-directed immunotherapies.

Extrinsic factors (*e.g.*, cell-cell interactions) and intrinsic ones (*e.g.*, cellular signaling proteins) contribute to T cell ligand discriminatory capability. A TCR typically binds to an agonist foreign–pMHC with a longer half-life than it does to a self–pMHC (20, 27–32). The TCR has higher ligand discrimination capability than B cell receptors (BCR), cytokine receptors, or other growth factor receptors (33). This discrimination capability relies on the intrinsic attributes of T cells to recognize self–pMHCs but not lead to activation, while recognition of foreign agonist–pMHCs should lead to potent T cell activation (34–38). Initial studies focused on the distinct characteristics of TCR binding kinetics toward foreign versus self–pMHC, such as the association on-rates and dissociation off-rates of the binding affinities between TCR and pMHC molecules (32), or the structural and biophysical change underlying a TCR:pMHC bound quality under forces to form a catch bonds (*e.g.*, force prolongs bond lifetime for TCR:agonist–pMHC) versus slip bonds (*e.g.*, force shortens bond lifetime for TCR:self–pMHC) (30, 39–41). However, those reported differences seem insufficient to account for a TCR’s high degree of specificity and sensitivity (29). Interestingly, by measuring the binding affinities of ultra-low TCR–pMHC affinities (those with a K_D_ in the range of ~1000 µM) at 37°C, a recent study showed that the level of TCR discrimination is lower than the level that was estimated by earlier work (42). Regardless, these models still need to be coupled with activation thresholds set by cellular signaling networks, thereby allowing a T cell to distinguish whether a given TCR–pMHC signal is below, or above, that activation threshold.

Thus, it is unclear how TCR ligand discrimination ability can be chemically encoded to control the T cell fate decision process and transform the TCR:pMHC signal to a digital activation outcome (1, 43), such as cytokine production or proliferation.

## Intracellular Signaling Pathways Control the Sensitivity and Selectivity of T Cell Ligand Discrimination Capability

**Figure 1:**
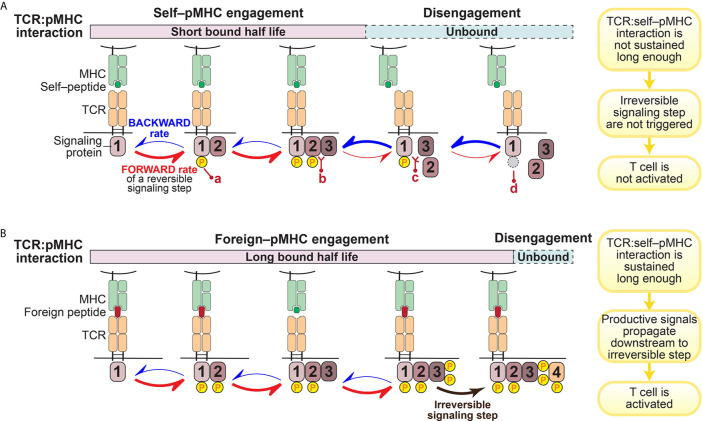
Illustration of kinetic proofreading model. The kinetic proofreading model envisions that TCR:pMHC engagement triggers a series of biochemical signaling steps, which eventually lead to activation of T cells. The series of biochemical signaling steps are reversible, allowing for TCR:pMHC disengagement to quickly restore signaling intermediates back to the initial resting stage. These reversible biochemical reactions could be phosphorylation (yellow circle; a)/dephosphorylation (gray circle; d), or protein-protein interaction (b)/dissociation (c). **(A)**. TCR:self–pMHC interaction is weak with a relatively short bound half life so that signaling does not propagate all the way downstream to an irreversible step before the TCR:pMHC dissociates. **(B)**. In contrast, the TCR interaction with foreign pMHC interaction is sufficiently long to reach a terminal irreversible step. Only when the TCR:pMHC interaction time is sustained long enough to engage all of the reversible kinetic proofreading steps and get to the key irreversible step will the T cell be activated.

A prevailing hypothesis is that the architecture of the intracellular signaling pathways downstream of the TCR engenders T cells with their remarkable sensitivity and selectivity for specific antigens (37, 38, 44). This is achieved through a *kinetic proofreading* mechanism that functions as a molecular timing device to set an activation threshold for T cells (45–47) (**Figure 1**).

The kinetic proofreading model was first proposed by Hopfield (47) and then adapted by McKeithan (45) to explain the remarkable selectivity of T cells. McKeithan envisioned that the TCR interaction with pMHC initiates a series of reversible biochemical reactions, such as phosphorylation, and these multiple steps create a time delay between the input (pMHC recognition) and the output (T cell activation) (45). If these signaling steps are rapidly reversible, the TCR:pMHC interaction must persist for a sufficient duration to allow signaling to reach a “competent state” and induce essentially irreversible signaling steps (such as the amplification of second messengers) in order to initiate successful T cell activation (45) (**Figure 1**). In other words, only those TCR:pMHC interactions with strong enough affinity, a long enough bound half-life, or a stable enough catch-bond formation can sustain the TCR proximal signaling long enough to overcome the temporal threshold to trigger a bona-fide activating signal by a T cell (**Figure 1B**). Built on the kinetic proofreading model, adaptation to intrinsic signaling events and modification of the signaling network (such as up-regulation of PD-1 or other negative regulators) can fine tune the reaction threshold in T cells (17, 48). Therefore, T cell ligand discrimination capability can potentially be engineered to change cellular outcomes.

A key prediction of the kinetic proofreading model is that through a series of reversible signaling steps, the TCR is triggered only when the last kinetic proofreading step is accomplished. The small differences in TCR–pMHC interaction– if coupled to a series of reversible and/or feedback loops that amplify small stimulatory signals and suppress excessive basal signaling– can lead to dramatically different cellular outcomes (38). In such a case, the more downstream in this TCR signal proofreading chain, the stronger difference in the level of signaling intermediates among TCR:pMHC of different half-lives, the greater discrimination is achieved. A recent study showed that it requires an approximate 2.8 sec of proofreading time delay and 2.67 biochemical steps to reach the estimated TCR discrimination capability (42). However, the specific key signaling steps where a small difference can enact a large change in functional outcome have not been fully identified or explored. In particular, it is not clear which early or intermediate signaling steps serve solely to pass along or amplify the signal, versus which terminal step(s) plays a more effectively digital role in terms of discriminating which ligands lead to activation versus those ligands that do not (31, 38, 49–53).

Many efforts have focused on understanding how the activities of protein kinases or phosphatases might provide the framework for the kinetic proofreading model. The Src family kinase (SFK) LCK and ζ chain-associated protein kinase 70 (ZAP-70), as two proximal and essential T cell specific kinases in TCR signal initiation, have attracted great interest regarding their supportive roles in kinetic proofreading.

LCK initiates the immediate signaling step after TCR:pMHC engagement (37, 54, 55). Functionally, LCK can phosphorylate the tyrosine residues of the immunoreceptor tyrosine-based activation motifs (ITAMs) in either the CD3 or ζ chains. Doubly phosphorylated ITAM motifs then create the docking sites for the tandem SH2 domains of ZAP-70 to bind and thereby be recruited to the engaged TCR complexes (56, 57). This docking step can be induced by the TCR binding to self–pMHC (56, 57). However, full release of ZAP-70 from its autoinhibitory conformation and its activation requires LCK to subsequently phosphorylate and activate the ITAM-bound ZAP-70 (58, 59). Afterwards, the activated ZAP-70 kinase phosphorylates tyrosine residues in adaptor proteins, including LAT and lymphocyte cytosolic protein 2 (SLP76), to promote the assembly of a LAT-based signalosome to diversify and amplify TCR signals. Of particular importance is the recruitment and activation of phospholipase C*γ*1 (PLC*γ*1) which is required for second messenger generation leading to calcium increases as well as Ras and protein kinase C (PKC) activation. Signaling beyond this point might be considered an irreversible (or a highly energy-consuming if it was to be reversed) signaling step since at least some T cell responses can be rapidly initiated subsequent to these events.

**Figure 2:**
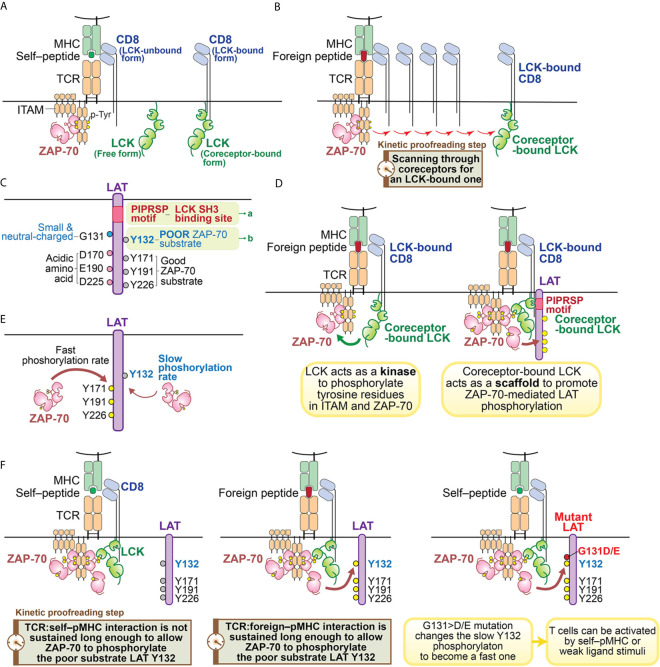
Illustration of kinetic proofreading steps. **(A)** Forms of LCK. LCK can exist as coreceptor-bound form or free form. The yellow circle represents the phosphorylated tyrosine residues. **(B)** oreceptor scanning model. After the TCR is engaged with pMHC, the TCR scans through coreceptors (CD4 or CD8) to find a coreceptor that is bound to active LCK. Engagement with an LCK-bound coreceptor may promote TCR sensitivity toward the weak ligand stimulation. The time involved to search for a LCK-bound coreceptor may function as a kinetic proofreading step. **(C)** Two unique features of LAT. Recent studies have revealed two unique features of LAT: 1) the PIPRSP motif in LAT can bind to SH3 domain of LCK (a); 2) LAT Y132 is a very poor substrate for ZAP-70 (b), because its preceding residue is a glycine. In contrast, other three tyrosine residues (Y171, Y191, Y226) are good ZAP-70 substrates because they all have acidic amino acid at the -1 positions. **(D)** Coreceptor-bound Lck has dual functions. LCK functions as both a kinase and a scaffold protein. As a kinase, LCK phosphorylates tyrosine residues of ITAM motifs in CD3 and ζ-chains. Doubly-phosphorylated ITAM motifs create the docking site for kinase ZAP-70. The recruited ZAP-70 is further phosphorylated by LCK and becomes activated. As a scaffold protein, the LCK SH2 domain binds to phosphorylated Y319 in ZAP-70 to promote continued LCK activity and ZAP-70 activity. LCK can also interact with LAT to actively recruit LAT to the pMHC engaged TCR complex. This active recruitment is mediated through LCK SH3 domain and LAT PIPRSP motif. The active recruitment of LAT to the proximity of pMHC-engaged TCR supports TCR sensitivity toward weak ligand stimulation. **(E)** LAT Y132 has a slow phosphorylation kinetics. On the contrary, Y171, Y191, Y226 in LAT have fast phosphorylation rates promoted by ZAP-70. **(F)** LAT Y132-PLC*γ*1 activation is a kinetic proofreading bottleneck. The adaptor protein LAT has five tyrosine residues, which are ZAP-70 substrates. Among these tyrosines, Y127, Y171, Y191 and Y226 all have either glutamate or aspartate at the -1 position, providing better binding to ZAP-70 substrate recognition site through an electrostatic selection mechanism. Therefore, these four tyrosine residues have faster phosphorylation kinetics mediated by ZAP-70. In contrast, LAT Y132 has a glycine residue at the -1 position, making Y132 a poor substrate to ZAP-70. This feature causes Y132 to have very slow phosphorylation kinetics relative to the other tyrosine residues in LAT. The slow phosphorylation of Y132 ensures that the PLC*γ*1 pathway is the last to be activated. PLC*γ*1 activation, a key event in T cell signaling events, may represent the last step in the chain of kinetic proofreading steps. The yellow circle represents the phosphorylated tyrosine residues and the gray circle represents the unphosphorylated tyrosine residues.

Like other SFKs, LCK has an SH3 domain, an SH2 domain, a tyrosine kinase domain and a C-terminal inhibitory tyrosine (60). However, the function of LCK is particularly noteworthy with regard to its roles in T cells. LCK’s unique N-terminal region anchors it to the plasma membrane by myristylation and palmitoylation, and allows it to associate with the coreceptors CD4 or CD8 (60–63) (**Figure 2A**). The coreceptor interaction is relatively weak, so LCK can also exist as a free, coreceptor-unbound form (64) (**Figure 2A**). Some recent studies suggest that the free form of LCK may be more mobile and more active than the coreceptor-bound form of LCK (38). However, coreceptor-bound LCK has some unique features which could play important roles in supporting TCR ligand discrimination (31, 39, 53, 65, 66) (**Figure 2B**). As suggested by a recent coreceptor scanning model (31), upon TCR recognition of a foreign pMHC, the engaged TCR may need to scan through many coreceptors to find one that is coupled with active LCK kinases to initiate signaling. The time involved in searching for the LCK-bound coreceptors is a potential kinetic proofreading step and can affect the sensitivity and magnitude of T cell responses (**Figure 2B**). This study also suggests coreceptor-bound LCK may display some function that is unique from the free LCK.

Indeed, each of the LCK structural domains are also suggested to play important roles in regulating TCR signaling. The LCK SH2 domain binds to phospho-Y319 of ZAP-70, promoting the open, active conformations of both LCK and ZAP-70 and sustaining coreceptor localization to the stimulated TCR (56, 59, 67, 68). Computational modeling also supports the notion that the interaction of LCK’s SH2 domain can increase the sensitivity of TCR-ligand recognition and the rapidity of TCR-induced activation (69). These observations on the SH2 domain of LCK, as well as previous studies that suggested an important role for the SH3 domain in TCR signaling and T cell development hinted that the SH3 domain of LCK might also contribute to downstream signaling in an important kinetic proofreading step (70–73).

We recently found a function of the LCK SH3 domain that had not previously been appreciated (49) (**Figures 2C, D** **)**. In examining the sequences of LAT among 41 mammalian species, we found that there are sixteen proline residues within the membrane-proximal region of LAT (from P33 to P95 in human LAT). The positions and frequencies of proline residues within this region are highly conserved in all mammalian LAT sequences that were examined. Yet the functionality of this region had not been appreciated. We found one particular proline motif in this region, PIPRSP (P80−P82−P85), interacted with the LCK SH3 domain and that this interaction was functionally important (**Figure 2C**).

Using a series of cellular experiments as well as molecular and biochemical approaches, we concluded that LCK, *via* its SH2 domain interacting with ZAP-70 phospho-Y319 and its SH3 domain by interacting with the identified LAT proline-rich domain, promotes ZAP-70-mediated LAT phosphorylation by bridging ZAP-70 to LAT (**Figure 2D**). This tri-protein interaction is likely to be a transient, low affinity interaction. We speculate that this higher order of protein-protein interaction might be illustrated by the following sequential intermolecular interactions: 1) the coreceptor-bound LCK uses its SH2 domain to bind to phospho-Y319 ZAP-70 thereby stabilizing its own catalytic function; 2) the coreceptor-bound LCK associates with the proline-rich PIPRSP motif in LAT *via* its SH3 domain; 3) in a coreceptor-bound form, LCK bridges these signal initiating proteins, ZAP-70 and LAT, to the engaged TCR:pMHC complexes.

This trimolecular protein interaction may facilitate weak TCR:pMHC interactions, especially those at the borderline of T cell activation thresholds, and contribute to the reversible kinetic proofreading signaling steps. In addition, this ZAP-70–LCK–LAT intramolecular coordination also supports TCR signal transduction in the proximity of engaged TCR:pMHC complexes. From the perspective of kinetic proofreading, this may be a key event to allow the internal signaling network to properly discriminate TCR interaction with an agonist or a self–pMHC. Thus, only the TCR:foreign–pMHC interaction would successfully propagate downstream signaling steps. Indeed, these data, combined with computational modeling, further revealed that this higher order of coreceptor-coupled protein-protein interaction is particularly important to endow TCRs with the sensitivity to detect and respond to weak ligands. Elimination of the proline-rich motif in LAT compromised TCR signaling and T cell development (49).

The kinetic proofreading model assumes that the signaling intermediates among the proofreading signaling steps can instantly reflect the status of TCR:pMHC interaction. Put differently, once the TCR is disengaged from pMHC, all the proofreading steps are quickly reversed (**Figure 1A**). Although such a reset process might exhibit some time delay and it remains elusive whether all signaling intermediates have to be reversed to the very initial or resting state, this assumption must be valid to a certain extent since it is essential for the kinetic proofreading model to work.

We envision that the quick reset of unsuccessful signaling can only be possible if all these signaling intermediates are in one physical complex, allowing for ligand unbinding from the TCR to quickly lead to the reversal of the signaling events (38). Thus, the scaffold function of coreceptor-bound LCK is unique as it enables LAT to be in the same complex with TCR:pMHC, ZAP-70 and LCK (38) (**Figures 2B, D**). These recent studies together support the model that coreceptor-bound LCK may have dual roles in supporting TCR signal initiation: as a kinase as well as a scaffold protein to orchestrate a distinct kinetic proofreading step in TCR signaling (**Figures 2B, D**).

In addition to the coreceptor-bound LCK data (31, 49), we and others also explored other signaling steps that might function as molecular timing devices in a similar manner (50–52). We focused our attention on another key tyrosine kinase involved in proximal TCR signaling, ZAP-70 (50, 74). Most ZAP-70 substrate phosphorylation sites are localized in two adaptor proteins, LAT and SLP76, which are known to be critically important for TCR-dependent T cell development and responses. This made LAT and SLP76 potentially interesting candidates with kinetic proofreading functions.

## The Scaffold Protein LAT Is a Unique Signaling Platform That Coordinates Multiple Signaling-Competent States at Differential Signaling Kinetic Rates

Scaffold proteins can also function within kinetic proofreading steps but have received less attention than kinases. Together with SLP76, LAT is a scaffold protein capable of amplifying T cell proximal signals upon TCR stimulation. LAT has a total of nine tyrosine residues and four of them have been intensively studied, including human LAT Y132, Y171, Y191 and Y226 (mouse LAT Y136, Y175, Y195 and Y235) (75, 76). The LAT tyrosine phosphorylation sites are important to initiate the assembly of higher order LAT complexes (77–79), which can become separated signaling microclusters in the membrane to facilitate downstream signaling, including actin reorganization, RAS/mitogen-activated protein kinase (MAPK) signaling, PKC activation, and calcium mobilization. LAT signalosome-induced signaling is also essential for most TCR induced signaling and transcriptional responses (79–81). Importantly, LAT signalosomes are also coupled to several negative regulators, such as thymocyte-expressed molecule involved in selection (THEMIS) (82–85), the Src homology region 2 domain-containing phosphatase 1 (SHP1) (86–88), and leucine-rich repeats and calponin homology domain containing 1 (LRCH1) (89).

## LAT G131 Is an Apparent TCR Signaling Bottleneck for TCR Activation-Induced Activation of PLCγ1 and Calcium Pathways

As adaptors in TCR signaling, LAT and SLP76 assemble signalosomes to link the ZAP-70-mediated “input” to downstream diverse pathways through individual key tyrosine substrates. Y132 in human LAT (Y136 in mouse) is especially important for the recruitment and activation of PLC*γ*1. Replacement of this key tyrosine with phenylalanine disrupted thymic development of T cells and TCR induced T cell activation (90–95). Importantly, unlike other key tyrosine residues that share redundant interacting partners, Y132 is the one and only tyrosine associated with PLC*γ*1 interaction and function. Activation of the PLC*γ*1 pathway increases the production of the second messengers diacylglycerol and inositol-1,4,5-triphosphate, and eventually increases PKC and Ras activation as well as elevation of the levels of intracellular Ca^2+^ concentration. Evidence provided by optogenetic controllers of TCR signaling has suggested calcium increases and diacylglycerol production are critically involved TCR kinetic proofreading (51, 52). These two studies indicate that the signaling steps that trigger the production of these second messengers may function as final signaling checkpoints to allow incoming signals to be properly proofread.

The phosphorylation of LAT Y132, leading to the recruitment and activation of PLC*γ*1 pathway, has some unique features. Biochemical and structural analysis of ZAP-70 revealed that the substrate binding site in its kinase domain is unusually enriched in basic residues (74). This feature creates a microenvironment that allows a tyrosine substrate with surrounding acidic amino acids, to serve as a good ZAP-70 substrate (74). Particularly important is a negatively charged residue at the -1 position (**Figure 2C**). Sequence analysis of the majority of known ZAP-70 substrates supported this model, including tyrosine residues in LAT (Y127, Y171, Y191 and Y226 in human LAT) and SLP76 (Y113, Y128, Y145 in human SLP76). These sites have either an aspartate or a glutamate residue preceding these known tyrosine substrates (**Figure 2C**). However, Y132 in human LAT (and Y136 in mouse LAT) is an exception (74, 96) (**Figure 2C**). Despite being a bona-fide and long known ZAP-70 substrate, Y132 has a neutral residue, glycine, at the 131 position (74) (**Figure 2C**). As a consequence, this glycine compromised Y132 phosphorylation by ZAP-70 compared to the other well-characterized tyrosine phosphorylation sites in LAT (**Figure 2E**). This observation was supported by an *in vitro* kinase assay which showed that replacement of glycine at 131 in LAT peptides with an aspartate or a glutamate substantially enhanced ZAP-70 kinase-mediated phosphorylation efficiency of LAT Y132 (50, 74). In an immunoblot analysis of LAT-deficient Jurkat cells reconstituted with G>D/E mutants of LAT or wild-type LAT, similar results were also observed (50). This slow phosphorylation of LAT Y132 is a feature that had been previously noted (96) but with an unclear basis or significance.

Interestingly, the simple substitution of an acidic residue, glutamate or aspartate, at the -1 position (*i.e.*, G131E or G131D) increased the phosphorylation rates of LAT Y132, the recruitment, phosphorylation and activation of PLC*γ*1, and the magnitude and the rate of calcium elevation (50, 97). The augmented LAT–PLC*γ*1–calcium pathway resulted in an increase in T cell responses (50). OT-I TCR transgenic CD8 T cells that were virally transduced with the gain-of-activity LAT mutants (G135>D or G135>E in mouse) acquired the ability to react with weak ligands or even self–pMHC which did not activate control CD8 T cells transduced with wild-type LAT (50) (**Figure 2F**). These hyper-sensitive OT-I TCR^+^ CD8 T cells expressing either G135>D or G135>E mutant LAT upregulated the expression of activation marker CD69 and increased their production of the proinflammatory cytokine IFN*γ* (50). A similar gain-of-activity from the ectopic expression of these two LAT mutants could also be observed in OT-II TCR^+^ CD4 T cells (50). Such enhanced responsiveness could have negative consequences *in vivo* since T cells expressing the G135>D/E LAT mutants might enable responses against self-peptides *in vivo* which could potentially result in autoimmunity or immunopathology.

The slow phosphorylation of Y132 in LAT appears to be a key and perhaps the most important rate-determining event in the kinetic proofreading process that assesses the quality of the TCR:pMHC interaction (**Figure 2F**). The slow phosphorylation rate of LAT Y132 is particularly interesting, for it is a unique kinetic proofreading event within a molecule which has five key tyrosine-based phosphorylation modifications which could allow multiple signaling-competent states to be achieved at different rates. However, each of the four other sites conform to preferred ZAP-70 substrates and the individual mutation of each of these other sites did not alter the sensitivity or magnitude of TCR response to pMHC (50). Thus, Y132 in LAT appears to a critically important step in kinetic proofreading, at least within the LAT protein. While Y132 appears to be a signaling bottleneck, it suggests the possibility that PLC*γ*1 activation is either the last step in the kinetic proofreading chain of events; or alternatively, the consequences of activation of the PLC*γ*1 pathway is the first signaling step not physically linked to the engaged TCR:pMHC complex. This model then further supports the importance of the LCK–LAT interaction within the stimulated TCR:pMHC complex for ligand discrimination, especially in the case of weak ligands.

The finding that nearly all tetrapods have a comparable glycine at the homologous position in LAT which is likely to lead to slow activation of PLC*γ*1 (50), but that other phosphorylation sites in LAT and even within SLP76 are better substrates for ZAP-70 (74), emphasizes the critical importance of Y132 as a critical timer to assess whether T cells should be fully activated to respond to potential threats in most warm-blooded organisms. Surprisingly, sequences conferring more rapid and efficient phosphorylation of LAT Y132 could be found in the LAT homologs of some cold-blooded animals, such as zebrafish. Thus, cold-blooded animals may require more efficient phosphorylation of the Y132 homologous sequence to ensure a strong PLC*γ*1 dependent responses in cold environments. Indeed, we showed that zebrafish thymocytes were less sensitive to cold temperatures than mammalian LAT (50). Expression of G131D in OT-I TCR^+^ Jurkat human T cell variants was able to endow these cells with the ability to respond to OVA stimulation at room temperature, whereas the wild-type glycine at position 131 in LAT prevented cells from responding at room temperature (50). Thus, our data suggested that the slow phosphorylation kinetics of human Y132 (and mouse Y136) in LAT evolved to be a poor site of phosphorylation in order to serve as a critical signaling step to control kinetic proofreading in mammalian T cells, whereas at least some cold-blooded animals require a more optimal site of phosphorylation at the Y132 homolog due to the constraint on phosphorylation imposed by cold temperature. These data offer insights into a novel mechanism to allow mammals to fine tune their T cell response threshold and contribute to T cell antigen discrimination.

## LAT Signalosome Maintains a Dynamic Interactome With the CD5 Signalosome and the CD6 Signalosome to Integrate Positive and Negative Regulatory Signals

Interestingly, the mutation of Y132 also led to TCR-independent lymphoproliferation of T cells (94, 98–102). This suggests PLC*γ*1 signal may serve a negative regulatory function to maintain the balanced cell signaling that leads to an appropriate T cell response. Thus, the LAT-assembled signalosome may multitask, with both positive and negative regulatory functions, within a complicated yet intricate signal transduction network.

A recent proteomic study revealed that the LAT signalosome exhibits dynamic time-dependent interaction relationships with the transmembrane receptors CD5- and CD6-assembled signalosomes (81). According to that study, the LAT signalosome mostly plays a dominant role as an activating signaling pathway node, as characterized by its ability to activate SOS-ERK and PLC*γ*1-NFAT pathways (81). However, unlike the LAT signalosome, the CD5 signalosome is enriched with many proteins with inhibitory functions, including Cbl-b and Ubash3A (81). Interestingly, the CD6-induced signalosome functions as a buffering zone, competing interacting proteins with either the inhibitory CD5 signalosome or with the stimulatory LAT signalosome. SLP76, GRB2, and GRB2 related adaptor protein 2 (GRAP2) can be associated with either the LAT signalosome or CD6 signalosome, whereas SOS, PLC*γ*1 and hematopoietic progenitor kinase 1 (HPK1) can only associate with the LAT signalosome (81). Competition of these shared interactors influences the relative ratio of active LAT signalosome versus that of the CD6 complex. For example, both CD6 and LAT interacts with SLP76; however, although SLP76 can bind to LAT through an intermediate protein GRB2 related adaptor protein 2 (GRAP2), SLP76’s SH2 domain is required for assembly with CD6 signalosome. This difference in the interaction mechanism allows SLP76 to only activate HPK1 when it resides in the LAT signalosome but not within the CD6 signalosome, thereby influencing the compositions and dynamics of these signalosomes. This study revealed that dynamic interactions among LAT-, CD5-, and CD6-signalosomes may engage different positive or negative feedback loop to further fine tune TCR signaling. Further work is needed to understand how such interactions are regulated during physiologic TCR responses to antigen.

## Conclusion and Future Perspectives

Recent advances in T cell-targeted immunotherapies, including the development of chimeric antigen receptor (CAR) T cells, have successfully leveraged our knowledge of T cell signal transduction and T cell activation to treat cancers and/or autoimmune diseases. Although CARs can elicit T cell effector functions, these artificial receptors appear to be much less efficient at detecting foreign antigens than genuine TCRs. Moreover, most CARs lack the ability to fully propagate signals through the canonical TCR signaling pathway and appear to bypass some key signaling steps (22, 103–105). For example, CARs are less efficient at recruiting the kinase ZAP-70, which is responsible for initiating TCR signaling and LAT phosphorylation, and they do not efficiently activate Ca^2+^ signaling. A recent study also shows that CAR-mediated signaling may bypass LAT signals. These differences between TCR- and CAR-mediated signaling may underlie common issues confounding CAR-T therapy, such as diminished *in vivo* persistence of the therapeutic cells. Thus, further studies engineering more responsive CAR-T cells by enhancing the ability of CARs to tap into intrinsic T cell signaling pathways might offer new insights into better control T cell sensitivity and selectivity.

## Author Contributions

W-LL and AW wrote the manuscript and approved the submitted version. All authors contributed to the article and approved the submitted version.

## Funding

AW was supported by NIH P01 AI091580 and 1R37AI114575.

## Conflict of Interest

The authors declare that the research was conducted in the absence of any commercial or financial relationships that could be construed as a potential conflict of interest.
